# Reversible Neurological Manifestations Preceding Biochemical Deterioration in Postpartum HELLP Syndrome—A Case Report and Literature Review

**DOI:** 10.1111/jch.70267

**Published:** 2026-05-04

**Authors:** Dario Colacurci, Stefano Raffaele Giannubilo, Laura Sarno, Caterina Fulgione, Laura Letizia Mazzarelli, Angelo Sirico, Maria Liberata Meccariello, Andrea Albanese, Dayana Quintili, Irene Lubinski, Mario Ascione, Carla Riccardi, Giorgia D'Apice, Mariarosaria Motta, Sara Iannantuoni, Chiara Murolo, Giuseppe Bifulco, Costantino Di Carlo, Andrea Ciavattini, Giuseppe Maria Maruotti

**Affiliations:** ^1^ Department of Public Health University of Naples Federico II Naples Italy; ^2^ Department of Clinical Sciences Polytechnic University of Marche Ancona Italy; ^3^ Department of Neurosciences, Reproductive Science and Dentistry University of Naples Federico II Naples Italy; ^4^ Mother and Child Department University Hospital Federico II Naples Italy; ^5^ Department of Woman, Child and General and Specialized Surgery, Obstetrics and Gynecology Unit University of Campania “Luigi Vanvitelli” Naples Italy

**Keywords:** headache, HELLP syndrome, posterior reversible encephalopathy, postpartum, seizure

## Abstract

Posterior reversible encephalopathy syndrome (PRES) is a rare but severe neurological complication associated with hypertensive disorders of pregnancy and HELLP syndrome. We report a postpartum case in which neurological manifestations preceded the full biochemical expression of HELLP syndrome. A 22‐year‐old primigravida was admitted at 36 + 0 weeks for premature rupture of membranes and fetal growth restriction, presenting with mild thrombocytopenia and elevated lactate dehydrogenase but normal liver enzymes. On postpartum day 1, she developed headache, visual disturbances, hypertensive crisis, and a generalized tonic–clonic seizure. Brain magnetic resonance imaging revealed bilateral cortical–subcortical T2/FLAIR hyperintensities consistent with PRES. At that time, laboratory findings were not yet diagnostic of HELLP syndrome; however, by postpartum day 3, severe thrombocytopenia and elevated transaminases confirmed the diagnosis. The patient was treated with magnesium sulfate, antihypertensive therapy, corticosteroids, and plasmapheresis, achieving complete clinical and radiological recovery. A narrative review of the literature indicated that neurological manifestations typically occur within 48 h postpartum. This case highlights that PRES may precede the full laboratory expression of HELLP syndrome and underscores the importance of early recognition, prompt neuroimaging, and multidisciplinary management to ensure favorable maternal outcomes.

## 1 Introduction

Hypertension during pregnancy is one of the main causes of maternal and perinatal morbidity and mortality [1]. Hypertensive disorders of pregnancy may be complicated by preeclampsia [2], eclampsia [3], and Hemolysis, Elevated Liver enzyme levels and Low Platelet count (HELLP) syndrome [4]. These conditions, including HELLP syndrome, may present with heterogeneous and sometimes atypical clinical features. In particular, HELLP syndrome is considered a severe variant of preeclampsia and affects approximately 0.2–0.8% of all pregnancies [5, 6]. Moreover, HELLP syndrome atypical forms, such as without hypertension or proteinuria, are numerous, leading to important diagnostic challenges [7]. In particular in the postpartum period, clinicians could have delayed diagnosis. Considering this, early recognition is important to prevent severe maternal complications [8, 9, 10, 11]. Additionally, a particularly challenging and misleading presentation is when neurological manifestations precede the biochemical deterioration of HELLP syndrome. This “neurology‐first” presentation may delay diagnosis and lead to misinterpretation as primary neurological disorders. Neurological complications are one of the most severe and potentially life‐threatening manifestations of hypertensive disorders of pregnancy. Posterior Reversible Encephalopathy Syndrome (PRES) [12] is a clinic‐radiological manifestation characterized by acute onset of symptoms, such as headache, visual disturbances, altered mental status, and seizures; PRES is generally associated with vasogenic edema predominantly affecting the parieto‐occipital regions [13]. Its pathophysiology involves endothelial dysfunction, blood–brain barrier disruption, and dysregulation of cerebral autoregulation [13]. In obstetric patients, PRES is reported in association with severe preeclampsia and eclampsia [14]. Emerging evidence suggests that systemic endothelial injury and microangiopathy in HELLP syndrome may contribute to early cerebral involvement [15]. While neurological symptoms are typically described after the diagnosis of HELLP syndrome, emerging reports suggest that central nervous system involvement may precede the full biochemical expression of the disease. This dissociation is associated with diagnostic delay and inappropriate management, with an increased risk of adverse maternal outcomes [16, 17, 18]. Early recognition is essential to have a proper diagnosis with a correct management, thanks to neuroimaging, in particular magnetic resonance imaging (MRI) [13, 14]. In this report, we describe a postpartum case in which neurological manifestations and MRI findings consistent with PRES preceded the biochemical deterioration of HELLP syndrome, and we provide a focused narrative review of similar cases, emphasizing pathophysiology, differential diagnosis, and clinical management.

## 2 Case Presentation

A 22‐year‐old healthy primigravida was admitted to our Obstetric Unit at 36 + 0 weeks of gestation because of premature spontaneous rupture of membranes (pPROM).

### 2.1 Pre‐Admission

The patient had no significant past medical history or preexisting hypertension. Clinicians had previously diagnosed isolated intrauterine growth restriction (IUGR) during prenatal ultrasound monitoring; fetal abdominal circumference was consistently below the 10th percentile; however, longitudinal umbilical artery Doppler velocimetry was within physiological limits.

### 2.2 At Admission

**FIGURE 1:**
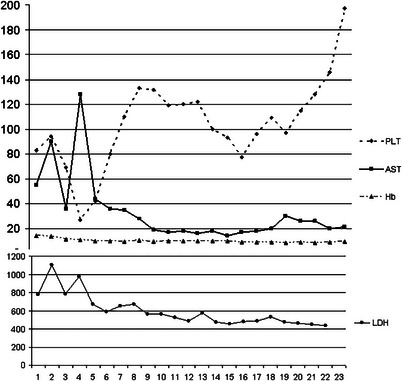
Blood sample values from the first day postpartum to the day of discharge. AST, Aspartate transaminase (U/L); Hb, Hemoglobin (g/dL); LDH, Lactate dehydrogenase (U/L); PLT, platelets count (109/L).

At the time of admission, the patient was asymptomatic and normotensive (115/75 mmHg). Laboratory screening revealed a mild, isolated thrombocytopenia (83 × 10^9^/L; reference range: 130–400×10^9^/L) and an elevated lactate dehydrogenase (LDH) (710 U/L; reference range: 227–450 U/L). Interestingly, serum creatinine, uric acid, and liver transaminases (AST/ALT) were within normal ranges. Figure 1 shows blood sample values from the first day postpartum to the day of discharge. Moreover, bedside 24‐h proteinuria was negative.

### 2.3 Delivery

The patient underwent an emergency cesarean section two days after admission, because of failure of induction of labor. The surgery was performed under spinal anesthesia without intraoperative complications. A male neonate weighing 2100 g (<fifth percentile) was delivered with an Apgar score of 8 at first minute and an Apgar score of 9 after 5 min.

### 2.4 Postpartum Day 1‐Neurological Onset

**FIGURE 2:**
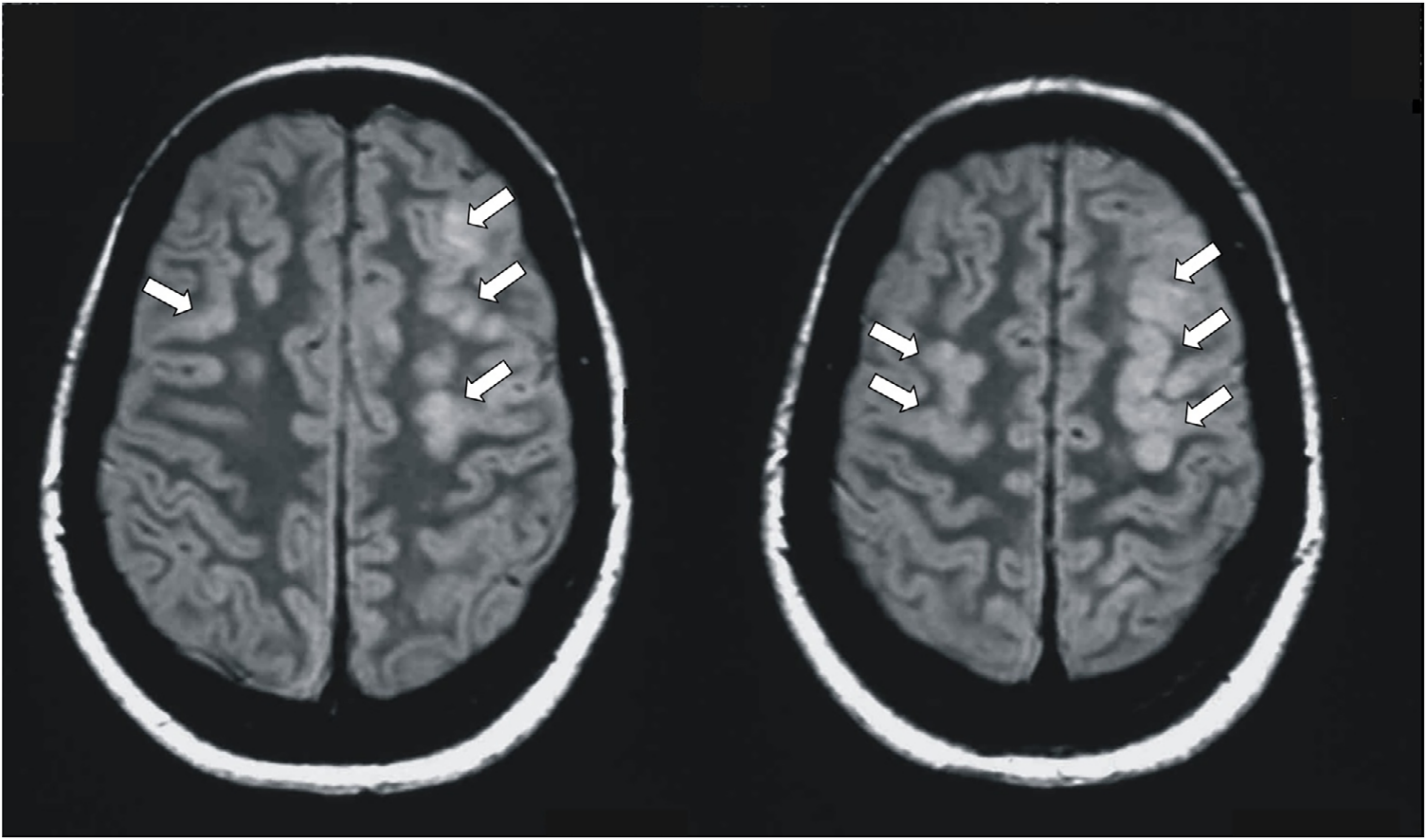
Brain MRI showing multiple, bilateral, and predominantly frontal and parietal, cortical–subcortical lesions, hypointense on T1‐weighted images and hyperintense on T2/FLAIR sequences.

On the first postpartum day, the patient's clinical and laboratory conditions changed significantly. She developed a rapid‐onset neurological syndrome characterized by profound lethargy, disorientation, and a persistent diffuse headache. This was followed by significant visual impairment, which the patient described as blurred vision and intermittent scotoma. Clinical assessment revealed a sudden hypertensive crisis, with blood pressure rising to 170/100 mmHg. While diagnostic imaging was being organized, the patient suffered from a generalized tonic‐clonic seizure lasting approximately 2 min. Instantly, intravenous magnesium sulfate (MgSO_4_) following the Zuspan regimen was administered, and the crisis ended. Immediate neuroimaging was performed to exclude intracranial hemorrhage or venous sinus thrombosis. MRI of the brain showed multiple bilateral, cortical, and subcortical lesions. These were predominantly localized in the frontal and parietal regions, showing T1‐hypointensity and marked T2/FLAIR‐hyperintensity (Fluid‐Attenuated Inversion Recovery). The radiological pattern was highly suggestive of PRES. Figure 2 shows brain MRI images with PRES radiological signs. Interestingly, laboratory tests immediately following the seizure showed a stable hemoglobin level (13.7 g/dL) and a slight improvement in the platelet count (94 × 10^9^/L), suggesting that the neurological event preceded the full biochemical manifestation of HELLP syndrome.

### 2.5 Postpartum Day 3‐Biochemical Deterioration and HELLP Diagnosis

Despite antihypertensive therapy with intravenous labetalol and seizure prophylaxis with MgSO_4_, the patient's biochemical profile deteriorated on the third postpartum day:

- Hemoglobin dropped to 10.7 g/dL.
- Platelet count dropped to 27 × 10^9^/L.
- AST and ALT rose to 128 U/L and 110 U/L respectively, while LDH increased further to 976 U/L.

These findings fulfilled the diagnostic criteria for HELLP syndrome.

### 2.6 Management and Recovery

Given the severity of the thrombocytopenia and the neurological involvement, a multidisciplinary team (Obstetrics, Neurology, and Hematology) opted for an aggressive management strategy. This included plasmapheresis (plasma exchange) and high‐dose corticosteroid therapy (dexamethasone 10 mg every 12 h). The patient showed a remarkable recovery under this regimen. Neurological symptoms began to decrease within 48 h. By the end of the first week, liver enzymes and platelet count showed a steady trend toward normal values. A follow‐up brain MRI performed 10 days later showed the complete disappearance of the signal abnormalities, confirming the reversible nature of the leukoencephalopathy.

### 2.7 Discharge

**FIGURE 3:**
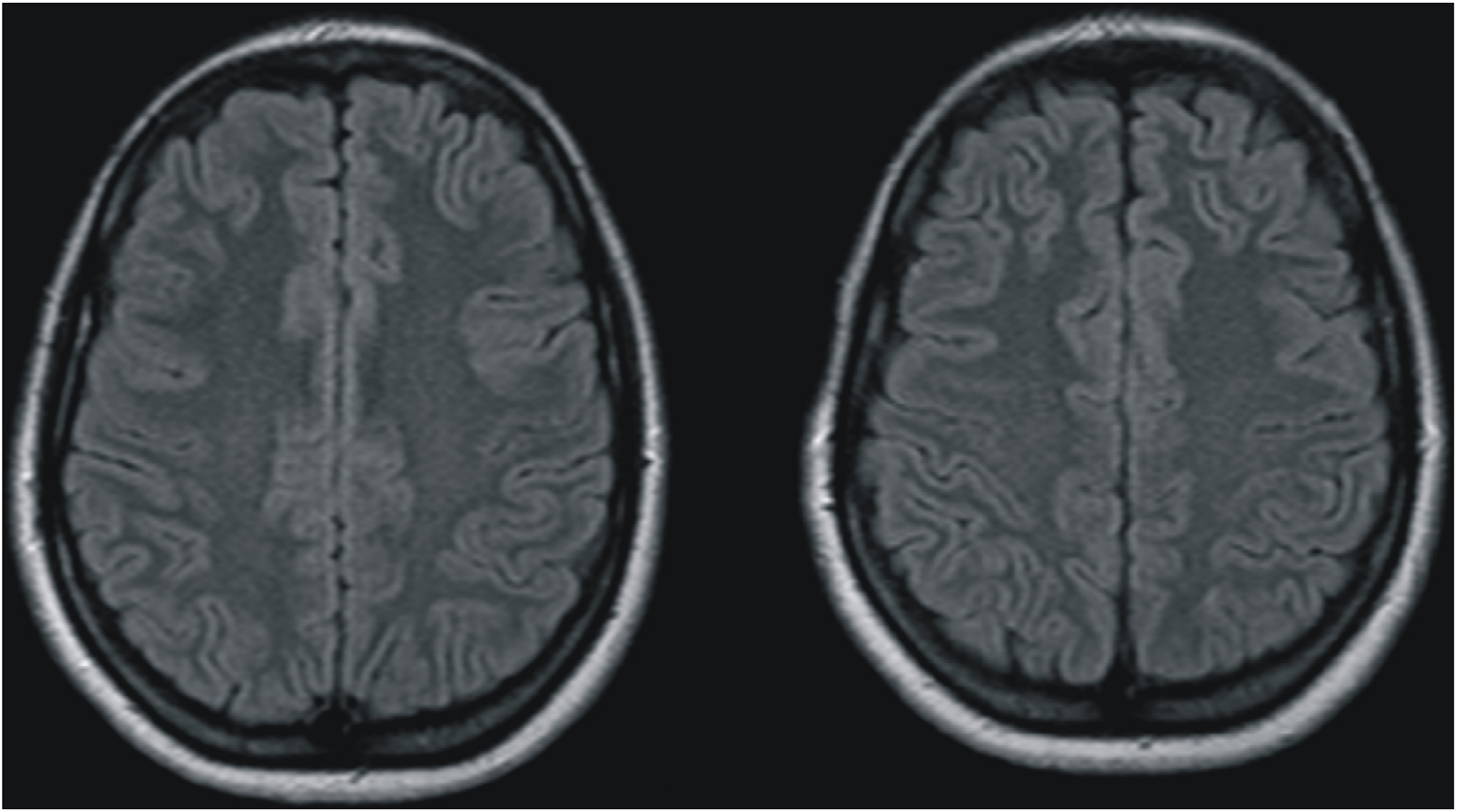
Follow‐up brain MRI showing the complete remission of signal abnormalities.

The patient was discharged on the 14th postpartum day in excellent clinical condition, with blood pressure well‐controlled on oral nifedipine, which was eventually interrupted. Figure 3 shows follow‐up brain MRI showing the complete remission of signal abnormalities.

## 3 Literature Review

A literature review was conducted to identify previously reported cases of postpartum HELLP syndrome complicated by neurological manifestations and PRES, using PubMed/MEDLINE and Scopus databases with the combinations of the following keywords: *“HELLP syndrome”, “posterior reversible encephalopathy syndrome”, “PRES”, “reversible posterior leukoencephalopathy syndrome”, “neurological complications”, “seizures”, “postpartum”, “pregnancy”, “preeclampsia”*, and *“eclampsia”*. Two authors independently screened titles/abstracts and full texts; disagreements were resolved by consensus. The search included articles published in English up to February 2026. Additional relevant studies were identified through manual screening of reference lists of selected publications. Eligible studies were case reports and case series describing women diagnosed with HELLP syndrome who developed neurological symptoms after delivery, supported by neuroimaging findings. Data regarding clinical presentation, laboratory abnormalities, neuroimaging features, therapeutic strategies, and clinical outcomes were extracted and summarized to provide contextual comparison with the present case. Given the descriptive nature of this review and the heterogeneity of available reports, no formal systematic selection process or quantitative synthesis was performed.

## 4 Results

Ten studies about HELLP syndrome complicated by postpartum neurological manifestations were identified and included in the analysis. Table 1 shows the summary of the clinical cases of HELLP syndrome and the neurological manifestations. All patients presented neurological symptoms after delivery, with onset between a few hours and 11 days postpartum. In most cases, neurological symptoms occurred in the first 48 h after delivery. The most common neurological manifestations included seizures, headache, visual disturbances, and altered mental status. Most cases had hypertension at the time of the neurological symptoms. Moreover, visual symptoms were found in almost all cases, in particular blurred vision, scotoma, amaurosis, and achromatopsia. Comorbidities were infrequent and were described in a minority of cases, principally consisting of preeclampsia or gestational hypertension. Fetal growth restriction (FGR) was observed in two cases. Laboratory abnormalities were consistent with the HELLP syndrome diagnostic criteria in all included cases. Table 2 shows the laboratory abnormalities, the neuroimaging features, the treatment, and the outcomes of the included studies. Thrombocytopenia was found to be the most significant laboratory abnormality. Additionally, platelet counts were usually below 100 × 10^9^/L with critical levels in some patients; elevated liver enzymes and increased LDH levels were observed in all cases, reflecting hepatic involvement and hemolysis. Anemia was described in a few patients, while renal dysfunction and electrolyte disturbances were less frequently documented. In some reports, including the present case, neurological symptoms preceded the onset of HELLP syndrome laboratory findings, defining a temporal dissociation between clinical and laboratory deterioration. At admission, the patient showed thrombocytopenia, elevated LDH, and FGR, while liver enzymes and proteinuria were still normal. Within 24 h postpartum she developed acute encephalopathy, visual symptoms and seizures with MRI findings consistent with PRES, whereas the classical HELLP biochemical triad became evident only on postpartum day 3.

**TABLE 1:** Summary of clinical cases of HELLP syndrome and neurological manifestations, including the temporal relationship between neurological onset and HELLP diagnosis.

Author	Year	Comorbidity	GA at HELLP syndrome diagnosis	GA at delivery	Symptoms and signs	Time of neurological symptoms and signs presentation	Temporal relationship (neurological symptoms vs. HELLP diagnosis)
Negro [19]	2005	None reported	Postpartum	39 w	Seizures, confusion, hypertension, visual loss	3 h postpartum	After delivery, HELLP diagnosed postpartum (likely simultaneous/early)
Peng [20]	2008	General edema, mild proteinuria	Postpartum	38 w	Hypertension, seizures with unconsciousness	5 h postpartum	After delivery, HELLP diagnosed postpartum (likely simultaneous/early)
Cozzolino [21]	2015	None reported	Postpartum	38 w	Headaches, epigastric pain, vomiting, hypertension	11 days postpartum	After HELLP diagnosis (delayed neurological onset)
Babahabib [17]	2015	None reported	Postpartum	38 w	Hypertension, headache, seizures	12 h postpartum	Likely simultaneous or shortly after HELLP diagnosis
Takahashi [22]	2017	None reported	28w + 6d	29 w	Hypertension, headache, epigastralgia, achromatopsia, IUGR	24 h postpartum	Likely simultaneous (HELLP diagnosed antepartum)
Vitucci [23]	2019	T3‐T4 spinal cord injury	Postpartum (3 h after delivery)	38 w	Hypertension, headache, vomiting, diplopia, perioral paresthesias, blurred vision	12 h after delivery	Likely simultaneous (HELLP diagnosed early postpartum)
Pantbalekundri [24]	2023	None reported	Postpartum	39 w	Postpartum hemorrhage, hypertension, seizures with unconsciousness, headache, pedal edema, choroiditis, retinal detachment	48 h postpartum	Likely simultaneous (HELLP diagnosed postpartum)
Medeiros [25]	2024	Depression, preeclampsia	35 w	35w	Hypertension, proteinuria, seizures, amaurosis, anisocoria	Intrapartum (second stage), worsening 1 h postpartum	Neurological symptoms at/around HELLP diagnosis (intrapartum/postpartum overlap)
Vuong [16]	2024	Severe preeclampsia	29w + 5d	30 w	Hypertension, tachypnea, SpO_2_ 93%, headache, epigastric pain, pulmonary edema, IUGR	At admission	Neurological symptoms at admission with severe preeclampsia/HELLP
Ždraljević [26]	2024	Gestational hypertension (from 30 w)	38 w	38 w	Hypertension, headache, blurred vision, tonic–clonic seizures	44 h postpartum	Likely simultaneous (HELLP diagnosed postpartum)

Abbreviations: d, days; GA, gestational age; Intrapartum, during labor; IUGR, intrauterine growth restriction; Postpartum, after delivery; SpO_2_, oxygen saturation; w, weeks.

**TABLE 2:** Laboratory findings, neuroimaging features, treatment, and outcomes of reported cases.

Author	Year	Laboratory tests	MRI images	Complications	Therapy	Time of resolution with discharge
Negro [19]	2005	Elevated transaminases; thrombocytopenia; Hb 9 g/dL; LDH 2053 U/L;	Diffuse supratentorial and infratentorial cortical–subcortical edema	None	PLEX; CS; AHT	14 days
Peng [20]	2008	AST 106 U/L; LDH: 854 U/L; PLT 80 × 10^9^/L;	Cortical and basal ganglia ischemic lesions	None	AHT; OSM; MgSO_4_	23 days
Cozzolino [21]	2015	AST 355 U/L; ALT 217 U/L; CPK 381 U/L; LDH 677 U/L; PLT 123 × 10^9^/L	Bilateral cerebellar cortical–subcortical vasogenic edema	None	AHT; MgSO_4_; CS	15 days
Babahabib [17]	2015	AST 525 U/L; ALT 214 U/L; Hb 7 g/dL; PLT 44×10^9/L	Bilateral parieto‐occipital cortical–subcortical T2/FLAIR hyperintensities	None	MgSO_4_; AED; AHT	5 days
Takahashi [22]	2017	AST 330 U/L; ALT 182 U/L; PLT 17 × 10^9^/L	Symmetric bilateral parieto‐occipital vasogenic edema	None	AHT; MgSO_4_; PLEX	28 days
Vitucci [23]	2019	AST 571 U/L; ALT 437 U/L; PLT 68 × 10^9^/L	Brainstem and cerebellar vasogenic edema with focal cytotoxic areas	None	AHT	11 days
Pantbalekundri [24]	2023	AST 153 U/L; ALT 136 U/L; hypoalbuminemia; Hb 10 g/dL; PLT 100 × 10^9^/L; LDH 5136 U/L	Bilateral parieto‐occipital and cerebellar vasogenic edema	None	AHT; DIU; RRT	Not reported
Medeiros [25]	2024	AST 100 U/L; ALT 95 U/L; sCr 1.8 mg/dL; PLT 36 × 10^9^/UL; hyperkalaemia; hypoalbuminemia	Asymmetric parieto‐occipital and cerebellar T2/FLAIR hyperintensities with focal diffusion restriction	Hysterectomy due to blood loss, seizures	PLEX; CS; AED	15 days
Vuong [16]	2024	AST 1325 U/L; ALT 600 U/L; LDH 2514 U/L; PLT 41 × 10^9^/L; hypoalbuminemia	Symmetric parieto‐occipital and basal ganglia T2/FLAIR hyperintensities	None	AHT; MgSO_4_; AED	5 days
Ždraljević [26]	2024	AST 227 U/L; ALT 213 U/L; Hb 9.3 g/dL; PLT 72 × 10^9^/L	Focal left occipital T2/FLAIR hyperintensity, no diffusion restriction	None	AHT	18 days

Abbreviations: AED, antiepileptic drugs; AHT, antihypertensive therapy; ALT, alanine aminotransferase; AST, aspartate aminotransferase; CPK, creatine phosphokinase; CS, corticosteroids; DIU, diuretics; DWI, diffusion‐weighted imaging; FLAIR, fluid‐attenuated inversion recovery; Hb hemoglobin; LDH, lactate dehydrogenase; MgSO_4,_ magnesium sulfate; MRI, magnetic resonance imaging; NA, not available; OSM, osmotherapy; PLEX, plasmapheresis; PLT, platelet count; RRT,: renal replacement therapy; sCr, serum creatinine; T2, T2‐weighted sequence.

Brain MRI was performed in all included cases and was considered a key diagnostic tool. The typical radiological finding was bilateral parieto‐occipital T2/FLAIR hyperintensities consistent with vasogenic edema, associated with PRES. Additional involvement of the cerebellum, brainstem, and basal ganglia was noted in some cases. A minority of patients showed superimposed cytotoxic edema associated with focal diffusion restriction. Ischemic lesions were noted in one of the cases. Follow‐up neuroimaging showed complete or near‐complete resolution of abnormalities. The management of the conditions was mainly based on blood pressure control, seizure prevention, and supportive care. Antihypertensive agents were administered to all patients. Most of the patients received magnesium sulfate for seizure prophylaxis or treatment. Antiepileptic agents were administered to patients with recurrent seizures. Severe cases with marked thrombocytopenia or neurological involvement were treated with immunomodulatory and extracorporeal therapies, such as corticosteroids and plasmapheresis. Renal replacement therapy and diuretics were required in patients with multiorgan dysfunction. A multidisciplinary approach involving obstetricians, neurologists, and intensivists was adopted in most reports. Finally, maternal outcomes were favorable. Neurological symptoms gradually resolved in all reported cases. Time to clinical resolution ranged from 5 to 28 days, with most patients achieving recovery within two weeks. Radiological resolution paralleled clinical improvement. Only one patient required surgical intervention because of severe obstetric hemorrhage. No maternal deaths were reported. The present case followed a similar course, with early neurological deterioration preceding laboratory worsening, rapid response to combined therapy, and complete clinical and radiological recovery.

## 5 Discussion

PRES is a clinic‐radiological disease associated with several medical conditions, such as hypertension, preeclampsia/eclampsia, immunosuppressive therapy, hemolytic uremic syndrome, acute glomerulonephritis, blood transfusion, intravenous immunoglobulin or erythropoietin administration, acute intermittent porphyria, intra‐abdominal neurogenic tumors, and severe hypercalcemia [14]. Clinically, it presents with acute neurological symptoms including headache, seizures, visual disturbances, and altered mental status [13]. Differential diagnosis of PRES in pregnancy and the puerperium may be challenging and includes stroke, cerebral venous thrombosis, encephalitis, and metabolic or demyelinating disorders. Misdiagnosis may result in delayed recognition with inappropriate treatment, potentially leading to irreversible neurological damage. In this context, particular attention should be paid to postpartum patients presenting with acute neurological symptoms, as HELLP syndrome may be initially overlooked when laboratory findings are not yet diagnostic. In our case, the patient developed a “neurology‐first” presentation of postpartum HELLP syndrome associated with PRES. MRI showed cortical and subcortical lesions with a distribution that was not limited to the typical parieto‐occipital regions and involved frontal and parietal areas [27]. Similar atypical patterns have been shown in other cases of HELLP associated with PRES [19, 20, 28], suggesting that lesion distribution may vary in this clinical context. PRES pathogenesis is not completely understood. Two main hypotheses are currently discussed: the first suggests that acute hypertension overwhelms cerebral autoregulation, leading to vasogenic edema; instead, the second emphasizes endothelial dysfunction and blood–brain barrier disruption, which may occur even in the absence of severe hypertension [29]. In obstetric patients, the high incidence of PRES may be explained by the vascular changes, such as altered vascular reactivity, impaired prostaglandin‐mediated vasodilation, and endothelial dysfunction; furthermore, the fluid accumulation and volume shifts may predispose patients to develop brain edema [30]. Importantly, the coexistence of systemic endothelial injury, microangiopathy, and coagulation abnormalities in HELLP syndrome may contribute to early blood–brain barrier disruption and cerebral edema, potentially explaining why neurological manifestations can precede the full biochemical expression of the disease. In our patient, pregnancy had been normotensive with IUGR, peripartum thrombocytopenia, and elevated LDH levels. The coexistence in pregnancy of IUGR and hypertensive disorders has traditionally been considered to reflect a common mechanism of uteroplacental insufficiency [31]. These findings support the hypothesis of an early systemic endothelial dysfunction preceding overt clinical and laboratory deterioration. Endothelial injury may represent a common pathogenic link between placental dysfunction and PRES, reinforcing the concept of a systemic disease affecting both maternal and cerebral vascular compartments. Schwartz et al. [32] suggested that LDH elevation may precede the development of brain edema and is not a consequence of hypertension alone. Moreover, specific markers, including red blood cell morphology and LDH levels, have been proposed as potential indicators of endothelial integrity and risk of progression to PRES. At admission, our patient also showed thrombocytopenia. Severe thrombocytopenia is a recognized marker of HELLP syndrome severity and may reflect disease progression [26], representing an additional marker of systemic endothelial injury. From a clinical perspective, early laboratory abnormalities such as thrombocytopenia, elevated LDH, and the presence of IUGR may represent warning signs (“red flags”) of underlying endothelial dysfunction and increased risk of neurological involvement. Based on our findings and the review of the literature, these features may help identify patients at higher risk of developing neurological complications, even in the absence of overt hypertension or diagnostic laboratory criteria for HELLP syndrome. Clinicians should consider these indicators as warning features that may justify closer monitoring and early neuroimaging when neurological symptoms occur. Management should focus on rapid blood pressure control, seizure prevention with magnesium sulfate, and timely treatment of HELLP syndrome. In severe cases, additional therapies such as corticosteroids and plasmapheresis may be required. In our case, early recognition and aggressive treatment resulted in complete clinical and radiological recovery, emphasizing the reversible nature of this syndrome when adequately managed. This highlights the importance of early diagnosis in preventing both neurological complications and inappropriate management strategies.

## 6 Conclusion

Postpartum HELLP syndrome complicated by PRES is a rare but clinically severe and potentially life‐threatening entity. Neurological manifestations may precede the full biochemical expression of HELLP syndrome and can occur even in patients who were previously normotensive, representing a “neurology‐first” presentation that may delay diagnosis and increase clinical risk. This presentation is particularly dangerous because it may be misdiagnosed as epilepsy, hypertensive encephalopathy, or other primary neurological disorders. Early multidisciplinary intervention, including blood pressure control, seizure management, and targeted therapy for HELLP syndrome, is essential to ensure complete clinical and radiological recovery. Therefore, in postpartum patients presenting with severe hypertension and neurological symptoms such as headache, seizure, or altered consciousness, HELLP syndrome should be promptly suspected even in the absence of diagnostic laboratory abnormalities. Early recognition of this pattern is critical to prevent delayed diagnosis and improve maternal outcomes.

## Author Contributions

**Andrea Albanese**: investigation, data collection, methodology. **Mario Ascione**: investigation, data collection, methodology. **Giuseppe Bifulco**: conceptualization, writing – review & editing, supervision, project administration, funding acquisition. **Costantino Di Carlo**: supervision, project administration, funding acquisition. **Andrea Ciavattini**: supervision, project administration, funding acquisition. **Dario Colacurci**: methodology, data curation, writing – original draft. **Giorgia D'Apice**: investigation, data collection, methodology. **Caterina Fulgione**: investigation, data collection, methodology. **Stefano Raffaele Giannubilo**: methodology, data curation, writing – original draft. **Laura Sarno**: conceptualization, methodology, writing – review & editing, supervision. **Sara Iannantuoni**: investigation, data collection, methodology. **Irene Lubinski**: investigation, data collection, methodology. **Giuseppe Maria Maruotti**: conceptualization, methodology, writing – review & editing, supervision. **Laura Letizia Mazzarelli**: investigation, data collection, methodology. **Maria Liberata Meccariello**: investigation, data collection, methodology. **Mariarosaria Motta**: investigation, data collection, methodology. **Dayana Quintili**: investigation, data collection, methodology. **Carla Riccardi**: investigation, data collection, methodology. **Angelo Sirico**: investigation, data collection, methodology.

## Funding

The authors have nothing to report

## Ethics Statement

This study was conducted in accordance with the ethical principles of the World Medical Association Declaration of Helsinki. According to institutional policies, ethical approval was not required for a single case report.

## Consent

Written informed consent was obtained from the patient for publication of this case report and any accompanying images.

## Conflicts of Interest

The authors declare no conflicts of interest.

## Permission to Reproduce Material from Other Sources

No material from other sources has been reproduced in this manuscript. All figures are original and created by the authors.

## Data Availability

The datasets generated and/or analyzed during the current study are available from the corresponding author on reasonable request.

## References

[1] Sunita , R. Kaushik , and P. K. Gaur , “Gestational Hypertension: A Contemporary Review of Epidemiology, Pathophysiology, and Therapeutic Approaches,” Current Hypertension Reviews 20 (2025): 117–126, 10.2174/0115734021312990241115045601. 39601171

[2] ACOG. Obstetrics & Gynecology 2019;133:1–1, 10.1097/AOG.0000000000003018.

[3] L. M. Eclampsia , “A Critical Pregnancy Complication Demanding Enhanced Maternal Care: A Review,” Medical Science Monitor 29 (2023): e939919, 10.12659/MSM.939919. 37415326 PMC10334845

[4] A. Petca , B. C. Miron , I. Pacu , et al., “HELLP Syndrome—Holistic Insight Into Pathophysiology,” Medicina (B Aires) 58 (2022): 326, 10.3390/medicina58020326. PMC8875732 35208649

[5] D. Mihu , N. Costin , C. M. Mihu , A. Seicean , and R. Ciortea , “HELLP Syndrome—A Multisystemic Disorder,” Journal of Gastrointestinal and Liver Diseases 16 (2007): 419–424. 18193124

[6] C. J. Saphier and J. T. Repke , “Hemolysis, Elevated Liver Enzymes, and Low Platelets (HELLP) Syndrome: A Review of Diagnosis and Management,” Seminars in Perinatology 22 (1998): 118–133, 10.1016/s0146-0005(98)80044-x. 9638906

[7] L. C. Poon , A. Shennan , J. A. Hyett , et al., “The International Federation of Gynecology and Obstetrics (FIGO) Initiative on Pre‐Eclampsia: A Pragmatic Guide for First‐Trimester Screening and Prevention,” International Journal of Gynecology & Obstetrics 145 (2019): 1–33, 10.1002/ijgo.12802. PMC6944283 31111484

[8] M. Palomo , M. Blasco , P. Molina , et al., “Complement Activation and Thrombotic Microangiopathies,” Clinical Journal of the American Society of Nephrology 14 (2019): 1719–1732, 10.2215/CJN.05830519. 31694864 PMC6895490

[9] W. Ye , H. Shu , Y. Wen , et al., “Renal Histopathology of Prolonged Acute Kidney Injury in HELLP Syndrome: A Case Series and Literature Review,” International Urology and Nephrology 51 (2019): 987–994, 10.1007/s11255-019-02135-z. 30989562

[10] L. Perronne , A. Dohan , P. Bazeries , et al., “Hepatic Involvement in HELLP Syndrome: An Update With Emphasis on Imaging Features,” Abdominal Imaging 40 (2015): 2839–2849, 10.1007/s00261-015-0481-1. 26099472

[11] G. G. Zeeman , “Neurologic Complications of Pre‐Eclampsia,” Seminars in Perinatology 33 (2009): 166–172, 10.1053/j.semperi.2009.02.003. 19464507

[12] J. P. Pirola , D. F. Baenas , N. R. Benzaquén , et al., “Posterior Reversible Leukoencephalopathy Syndrome: Case Series and Review of the Literature,” Reumatologia Clinica 16 (2020): 169–173, 10.1016/j.reuma.2018.04.006. 29859809

[13] J. E. Fugate and A. A. Rabinstein , “Posterior Reversible Encephalopathy Syndrome: Clinical and Radiological Manifestations, Pathophysiology, and Outstanding Questions,” Lancet Neurology 14 (2015): 914–925, 10.1016/S1474-4422(15)00111-8. 26184985

[14] J. D. Triplett , M. A. Kutlubaev , A. G. Kermode , and T. Hardy , “Posterior Reversible Encephalopathy Syndrome (PRES): Diagnosis and Management,” Practical Neurology 22 (2022): 183–189, 10.1136/practneurol-2021-003194. 35046115

[15] M. I. Sarbu and N. Sarbu , “Central PRES (Posterior Reversible Encephalopathy Syndrome) in HELLP Syndrome,” Internal and Emergency Medicine 14 (2019): 617–618, 10.1007/s11739-018-2003-y. 30519918

[16] A. D. B. Vuong , X. T. T. Pham , and P. N. Nguyen , “Posterior Reversible Encephalopathy Syndrome (PRES) on the Second Postpartum Day: Learning Experience From a Case Report and Literature Review,” International Journal of Emergency Medicine 17 (2024): 118, 10.1186/s12245-024-00707-0. 39251910 PMC11386115

[17] M. A. Babahabib , I. Abdillahi , F. Kassidi , J. Kouach , D. Moussaoui , and M. Dehayni , “Posterior Reversible Encephalopathy Syndrome in Patient of Severe Preeclampsia With Hellp Syndrome Immediate Postpartum,” Pan African Medical Journal 21 (2015), 10.11604/pamj.2015.21.60.5546. PMC4564409 26405496

[18] J.‐D. Rho , Y.‐H. Kim , J.‐H. Shin , and T. K. Kim , “Case Report: A Case of Posterior Reversible Encephalopathy in Postpartum Preeclampsia,” Medicine 102 (2023): e36023, 10.1097/MD.0000000000036023. 38013383 PMC10681536

[19] A. Negro , G. Zuccoli , G. Regolisti , S. Mastrangeli , and E. Rossi , “Reversible Posterior Leukoencephalopathy Associated With Postpartum HELLP Syndrome,” European Journal of Internal Medicine 16 (2005): 291–293, 10.1016/j.ejim.2004.11.010. 16084357

[20] W.‐X. Peng , M. Nakaii , T. Matsushima , and H. Asakura , “Atypical Case of Reversible Posterior Leucoencephalopathy Syndrome Associated With Puerperal HELLP Syndrome,” Archives of Gynecology and Obstetrics 278 (2008): 269–271, 10.1007/s00404-008-0578-7. 18247035

[21] M. Cozzolino , C. Bianchi , G. Mariani , L. Marchi , M. Fambrini , and F. Mecacci , “Therapy and Differential Diagnosis of Posterior Reversible Encephalopathy Syndrome (PRES) During Pregnancy and Postpartum,” Archives of Gynecology and Obstetrics 292 (2015): 1217–1223, 10.1007/s00404-015-3800-4. 26122264

[22] H. Takahashi , T. Matsubara , S. Makino , K. Horie , and S. Matsubara , “Color Vision Abnormality as the Sole Manifestation of Posterior Reversible Encephalopathy due to Post‐Partum HELLP Syndrome,” Journal of Obstetrics and Gynaecology Research 43 (2017): 592–594, 10.1111/jog.13241. 28109137

[23] A. Vitucci , A. Lojacono , E. Gatti , F. Prefumo , and N. Fratelli , “Atypical Presentation and Imaging Features of Postpartum Posterior Reversible Encephalopathy Syndrome,” Journal of Obstetrics and Gynaecology (Lahore) 39 (2019): 412–414, 10.1080/01443615.2018.1474188. 30226402

[24] N. Pantbalekundri , S. Mathurkar , N. Acharya , S. Kumar , and A. S. Choroidopathy , “Retinal Detachment: A Rare Sighting in a Case of Postpartum Hemorrhage Presenting With Posterior Reversible Encephalopathy Syndrome,” Cureus (2023), 10.7759/cureus.50731. PMC10792348 38234958

[25] R. Pinto Medeiros , M. Ruão , P. Vita , R. Monte , and A. Marinho , “Postpartum HELLP Syndrome Associated With Posterior Reversible Encephalopathy Syndrome,” European Journal of Case Reports in Internal Medicine 12 (2024), 10.12890/2024_005019. PMC11716313 39790849

[26] M. Ždraljević , A. Pejović , B. Jocić‐ Pivač , M. Budimkić , D. R. Jovanović , and M. Mijajlović , “Rare Association of Posterior Reversible Encephalopathy Syndrome (PRES) With Hemolysis, Elevated Liver Enzymes and Low Platelets (HELLP) Syndrome—A Case Report and Review of the Literature,” Heliyon 10 (2024): e40915, 10.1016/j.heliyon.2024.e40915. 39802606 PMC11724767

[27] J. B. Miller , K. Suchdev , N. Jayaprakash , et al., “New Developments in Hypertensive Encephalopathy,” Current Hypertension Reports 20 (2018): 13, 10.1007/s11906-018-0813-y. 29480370

[28] E. Marano , N. Scuteri , G. Vacca , and G. Orefice , “HELLP Syndrome With Reversible Posterior Leukoencephalopathy,” Neurological Sciences 24 (2003): 82–84, 10.1007/s100720300078. 12827545

[29] O. Demirtaş , F. Gelal , B. D. Vidinli , L. O. Demirtaş , E. Uluç , and A. Baloğlu , “Cranial MR Imaging With Clinical Correlation in Preeclampsia and Eclampsia,” Diagnostic and Interventional Radiology 11 (2005): 189–194. 16320222

[30] A. B. Singhal , “Reversible Cerebral Vasoconstriction Syndrome: A Review of Pathogenesis, Clinical Presentation, and Treatment,” International Journal of Stroke 18 (2023): 1151–1160, 10.1177/17474930231181250. 37246916

[31] G. J. Burton and E. Jauniaux , “Pathophysiology of Placental‐Derived Fetal Growth Restriction,” American Journal of Obstetrics and Gynecology 218 (2018): S745–S761, 10.1016/j.ajog.2017.11.577. 29422210

[32] R. B. Schwartz , S. K. Feske , J. F. Polak , et al., “Preeclampsia‐Eclampsia: Clinical and Neuroradiographic Correlates and Insights Into the Pathogenesis of Hypertensive Encephalopathy,” Radiology 217 (2000): 371–376, 10.1148/radiology.217.2.r00nv44371. 11058630

